# Hypoxia and vitamin D differently contribute to leptin and dickkopf-related protein 2 production in human osteoarthritic subchondral bone osteoblasts

**DOI:** 10.1186/s13075-014-0459-3

**Published:** 2014-10-14

**Authors:** Béatrice Bouvard, Elie Abed, Mélissa Yéléhé-Okouma, Arnaud Bianchi, Didier Mainard, Patrick Netter, Jean-Yves Jouzeau, Daniel Lajeunesse, Pascal Reboul

**Affiliations:** UMR 7365 CNRS-Université de Lorraine, IMoPA, Biopôle de l’Université de Lorraine, Campus Biologie-Santé, 9 Avenue de la Forêt de Haye- CS 50184, 54505 Vandoeuvre lès Nancy, Cedex France; Present address: Service de Rhumatologie, Centre Hospitalier Universitaire, 4 rue Larrey, 49933 Angers cedex 9, France; Unité de Recherche en Arthrose, Centre de Recherche de l’Université de Montréal (CR-CHUM), 900, rue Saint-Denis, Montréal, QC Canada H2X 0A9; Centre Hospitalier Universitaire, 29 Avenue du Maréchal de Lattre de Tassigny, 54000 Nancy, France

## Abstract

**Introduction:**

Bone remodelling and increased subchondral densification are important in osteoarthritis (OA). Modifications of bone vascularization parameters, which lead to ischemic episodes associated with hypoxic conditions, have been suspected in OA. Among several factors potentially involved, leptin and dickkopf-related protein 2 (DKK2) are good candidates because they are upregulated in OA osteoblasts (Obs). Therefore, in the present study, we investigated the hypothesis that hypoxia may drive the expression of leptin and DKK2 in OA Obs.

**Methods:**

Obs from the sclerotic portion of OA tibial plateaus were cultured under either 20% or 2% oxygen tension in the presence or not of 50 nM 1,25-dihydroxyvitamin D_3_ (VitD_3_). The expression of leptin, osteocalcin, DKK2, hypoxia-inducible factor 1α (Hif-1α) and Hif-2α was measured by real-time polymerase chain reaction and leptin production was measured by enzyme-linked immunosorbent assay (ELISA). The expression of Hif-1α, Hif-2α, leptin and DKK2 was reduced using silencing RNAs (siRNAs). The signalling pathway of hypoxia-induced leptin was investigated by Western blot analysis and with mitogen-activated protein kinase (MAPK) inhibitors.

**Results:**

The expression of leptin and DKK2 in Obs was stimulated 7-fold and 1.8-fold, respectively (*P* <0.05) under hypoxia. Interestingly, whereas VitD_3_ stimulated leptin and DKK2 expression 2- and 4.2-fold, respectively, under normoxia, it stimulated their expression by 28- and 6.2-fold, respectively, under hypoxia (*P* <0.05). The hypoxia-induced leptin production was confirmed by ELISA, particularly in the presence of VitD_3_ (*P* <0.02). Compared to Obs incubated in the presence of scramble siRNAs, siHif-2α inhibited VitD_3_-stimulated leptin mRNA and protein levels by 70% (*P* =0.004) and 60% (*P* <0.02), respectively, whereas it failed to significantly alter the expression of DKK2. siHif-1α has no effect on these genes. Immunoblot analysis showed that VitD_3_ greatly stabilized Hif-2α under hypoxic conditions. The increase in leptin expression under hypoxia was also regulated, by p38 MAPK (*P* <0.03) and phosphoinositide 3-kinase (*P* <0.05). We found that the expression of leptin and DKK2 were not related to each other under hypoxia.

**Conclusions:**

Hypoxic conditions via Hif-2 regulation trigger Obs to produce leptin, particularly under VitD_3_ stimulation, whereas DKK2 is regulated mainly by VitD_3_ rather than hypoxia.

## Introduction

Osteoarthritis (OA) is considered a systemic and heterogeneous disease. The endpoint of OA is cartilage degradation, which is associated with and/or preceded by subchondral bone alterations [1] and synovial membrane inflammation [2]. The chronological events of these phenomena are still being debated in the literature, although recent developments emphasize the pathophysiological role of subchondral bone [3]. In OA, it would appear that the extracellular matrix of both cartilage and subchondral bone are altered [4,5]. Early-stage OA increases remodelling and bone loss, whereas late-stage OA decreases remodelling and increases subchondral densification. Both stages are important components of the pathogenic process that leads to OA lesions [3]. In regard to subchondral bone changes, modifications of vascularization parameters have been suspected. The subchondral region of long bones is particularly vascularised, indicating high nutrient supply [6] not only for bone cells but also for delivering nutrients to the chondrocytes of the deep layers of articular cartilage [7]. Therefore, the epiphyses are at risk of circulatory insufficiency. Although vascularization of the subchondral bone plate seems to increase during OA progression [8,9] and there are occurrences of numerous microchannels through the osteochondral plate [10], the literature contains evidence of blood flow disturbances in this region. For instance, venous stasis [11] was noted a long time ago and more recently was confirmed with dynamic contrast-enhanced magnetic resonance imaging (MRI) [12] and positron electron transfer [13]. Venous stasis sometimes leads to intraosseous hypertension in patients with OA [11]. Furthermore, similar blood flow disturbances were demonstrated in the subchondral bone medial plateau of guinea pigs, which develop spontaneous knee OA [14]. In this model, the observation of outflow obstruction preceding bone and cartilage OA lesions lends support to a key role of abnormal vascularization of OA bone tissues. These perfusion abnormalities have also been associated with osteonecrosis and bone marrow lesions [12], which are dynamic episodes visualised by MRI often predictive of progressive OA for these patients [15-18]. Hence, this altered OA subchondral bone vascularization leads to ischemic episodes associated with hypoxic conditions [11,19,20]. Interestingly, the presence of carbonic anhydrase IX, a recognized hypoxia biomarker, has recently been demonstrated in osteoarthritic bone [21]. Hypoxia has been shown to drive several pathways in bone, such as growth [22], regeneration and disease [23,24], as well as the maintenance of haematopoiesis [25]. Recently, Chang et al. demonstrated that hypoxia induced vascular endothelial growth factor (VEGF) expression as well as other genes involved in bone vasculature in OA osteoblasts (Obs) [21]. In addition, they suggested that hypoxia modifies the OA Ob phenotype. Key mediators—namely, hypoxia-inducible transcription factors (Hifs)—play a vital role during these hypoxia. Hif heterodimers are composed of an oxygen-sensitive α-subunit and a constitutively expressed β-subunit. Hif-1α and Hif-2α are the best characterized α isoforms [26]. During hypoxia, an accumulated Hif-α protein translocates to the nucleus and forms a dimer with Hif-β.

Among several factors potentially involved in OA pathogenesis, leptin and dickkopf-related protein 2 (DKK2) are good candidates [27-31], and their local production by Obs isolated from the subchondral bone seems to be important [29,31,32]. Leptin levels in the synovial fluid are greater in OA patients than that in healthy people, and this adipokine exerts a proinflammatory effect in synovial fibroblasts [33]. The level of leptin also increases in cartilage with the severity of OA as well as in subchondral bone [29,34]. In fact, leptin might act as a proinflammatory factor on cartilage metabolism and exerts a catabolic effect on OA joints [35]. Interestingly, members of our collaborative group previously reported that DKK2 is increased fourfold in OA Obs and that it also inhibits Wnt/β-catenin signalling, suggesting that DKK2 participates in the altered phenotype of OA Obs and to the reduced mineralization [31]. The regulation of leptin gene by hypoxia seems to be cell-specific [36-38] or cell environment–dependent [39,40], whereas DKK2 has not yet been described as a potential target of Hifs. Therefore, in the present study, we investigated this hypothesis in experiments using human OA Obs isolated from the subchondral bone plate.

## Methods

### Patients and clinical parameters

Tibial plateaus were obtained from patients with OA who were undergoing total knee replacement surgery. A total of 14 patients (age 69.1 ± 8.7 years; BMI 31.4 ± 9.6; 10 women and 4 men) who had OA according to the recognized clinical criteria of the American College of Rheumatology were included [41]. All specimen collection and all procedures were approved by the ethics committee of the Nancy University Hospital (CHU) (agreement UF 9607-CPRC 2005) and conducted in conformity with the Declaration of Helsinki principles. Written informed consent was obtained from all participants as mentioned elsewhere [42].

### Preparation of primary subchondral bone cell cultures

Isolation of the subchondral bone plate and preparation of Ob cultures were performed using medial and lateral tibial plateaus, where bone sclerosis is observed as described previously [43]. Cells were passaged once at 25,000 cells/cm^2^ and grown for 5 days in Dulbecco’s modified Eagle’s medium/F12 containing 10% foetal bovine serum (FBS) and a 1% penicillin-streptomycin mixture. Confluent cells were then preconditioned for 24 hours with the same medium containing 0.5% FBS and then incubated in the presence of 50 nM 1,25-dihydroxyvitamin D_3_ (VitD_3_; Sigma-Aldrich, St-Quentin Fallavier, France) or of the vehicle (ethanol 96%, 1 μl/ml) under normoxia (20% O_2_) or hypoxia (2% O_2_) for different periods of time as specified in the experiments. Supernatants were collected at the end of the incubation period and kept at −20°C prior to assays.

### RT-PCR assays

Total RNA was extracted from Obs using the RNeasy Mini Kit (QIAGEN, Courtaboeuf, France) according to the manufacturer’s instructions. Gene expression was analysed by quantitative real-time PCR (StepOnePlus Real-Time PCR System; Applied Biosystems, St-Aubin, France) using iTaq Universal SYBR Green Supermix (Bio-Rad Laboratories, Marnes la Coquette, France) according to the manufacturer’s protocol. The gene-specific primer pairs are given in Table 1. *RP29* served as a housekeeping gene.

**Table 1:** **Oligonucleotides used for real-time RT-PCR** ^**a**^

**Gene**		**Sequence 5′-3′**	**Gene ID**
*RP29*	S	5′-GGG TCA CCA GCA GCT CGA GA-3′	NM_001032.3
	AS	5′-CAG ACA CGA CAA GAG CGA GA-3′	
*Leptin*	S	5′-GGC TTT GGC CCT ATC TTT TC-3′	NM_000230.2
	AS	5′-GGA TAA GGT CAG GAT GGG GT-3′	
*DKK2*	S	5′-GGG TTT TGC TGT GCT CGT-3′	NM_014421.2
	AS	5′-TGG CTT TGG AGG AGT AGG TG-3′	
*Hif-1*	S	5′-GAA AGC GCA AGT CCT CAA AG-3′	NM_181054.2
	AS	5′-TGG GTA GGA CAT GGA GAT GC-3′	
*Hif-2*	S	5′-TTG-ATG-TGG-AAA-CGG-ATG-AA-3′	NM_001430.4
	AS	5′-GGA-ACC-TGC-TCT-TGC-TGT-TC-3′	
*Osteocalcin*	S	5′-CAT-GAG-AGC-CCT-CAC-A-3′	NM_199173.4
	AS	5′-AGA-GCG-ACA-CCC-TAG-AC-3′	
*VEGFα*	S	5′-TGG-GCC-TTG-CTC-AGA-GCG-GA-3′	NM_001171629.1
	AS	5′-GCC-TTG-CAA-CGC-GAG-TCTGT-3′	
*TGFβ1*	S	5′-GCG-TGC-TAA-TGG-TGG-AAA-C-3′	NM_000660.5
	AS	5′-GCT-GAG-GTA-TCG-CCA-GGA-A-3′	
*Sirt1*	S	5′-GCT-GGA-ACA-GGT-TGC-GGG-AA-3′	NM_012238.4
	AS	5′-GGG-CAC-CTA-GGA-CAT-CGA-GGA-3′	

S, sense; AS, Antisense; DKK2, Dickkopf-related protein 2; Hif-2, Hypoxia-inducible factor 2; TGF-β1, Transforming growth factor β1; VEGFα, Vascular endothelial growth factor α.

### Western blot analysis

Cell extracts were loaded onto 4–20% SDS-PAGE precast gels (Bio-Rad Laboratories), and Western immunoblot analysis and signal detection were performed as previously described [44]. As primary antibodies, we used a mouse anti-Hif-2α monoclonal antibody at a dilution of 1:1,000 (Thermo Scientific, Rockford, IL, USA), a rabbit anti-Hif-1α polyclonal antibody at a dilution of 1:1,000 (Novus Biologicals Interchim, Montluçon, France) and a rabbit anti-actin antibody at a dilution of 1:8,000 (Sigma-Aldrich). Anti-mouse and anti-rabbit horseradish peroxidase–conjugated immunoglobulin G secondary antibodies were used at a dilution of 1:10,000 (Thermo Scientific).

### Inhibition of Hif-1α and Hif-2α expression by RNA silencing

Silencing RNAs (siRNAs) were obtained from Eurogentec (Liège, Belgium), and we followed the manufacturer’s directions for their preparation. OA Obs were split into 24-well plates at 25,000 cells/cm^2^ to obtain 50% confluence at the time of transfection. siHif-2α, siHif-1α or scramble siRNA (siScr; basal condition) was added to OA Obs at a final concentration of 10 nM in the presence of 2 μl of INTERFERin™ reagent (Polyplus Transfection, Strasbourg, France). After 24 hours, cells were starved with medium containing 0.5% FBS for 24 hours and then treated with 50 nM VitD_3_ or ethanol under hypoxic conditions for 6, 24 or 48 hours. Conditioned media were collected at the end of the incubation period and kept at −20°C prior to assays. Cells were harvested in either Buffer RLT Plus lysis buffer with β-mercaptoethanol (QIAGEN) for RNA extraction or in Laemmli 1× buffer for protein extraction. These experiments were reproduced with Obs from 5 different patients; (n= 5). Experiments with siRNAs targeting leptin or DKK2 were performed for a 48-hour period under similar hypoxic conditions. To evaluate Hif-1 activity, OA Obs split in 24-well plates were transfected with 500 ng of a Hif-1 response element Renilla luciferase reporter construct (SwitchGear Genomics, Carlsbad, CA, USA) in the presence of a polycationic transfection reagent (ExGen 500; Euromedex, Strasbourg, France) according to the manufacturer’s recommendations (in triplicates for each condition; *n* =4). Renilla luciferase activity was measured after 36-hour incubation of Obs in hypoxic conditions.

### Inhibition of p40/p42 MAPK, p38 MAPK and phosphoinositide 3-kinase

OA Obs (*n* =5) were treated with p40/p42 inhibitor (10 μM PD98059; Calbiochem, Darmstadt, Germany), p38 inhibitor (5 μM SB203580; Calbiochem) or phosphoinositide 3-kinase (PI3K) inhibitor (5 μM LY294002; Calbiochem), or with vehicle (1 μl/1,000 μl dimethyl sulphoxide), under normoxia 1 hour before addition of 50 nM VitD_3_ or ethanol and incubation under hypoxia for 48 hours. Conditioned media were collected at the end of the incubation period for evaluation of leptin production (see next subsection), and cells were lysed in 0.5% SDS for protein quantitation by bicinchoninic acid assay.

### Evaluation of leptin production

Leptin was evaluated in Obs-conditioned media using a human Quantikine immunoassay (R&D Systems, Minneapolis, MN, USA). The sensitivity of the assay is 7.8 pg/ml, and the intra-assay precision is 3.2 ± 0.2%.

### Statistical analysis

Data are expressed as mean ± SEM. Statistical analyses were performed using either one-way analysis of variance followed by Dunnett’s multiple comparisons *post hoc* test or a *t*-test using GraphPad Prism 5 (GraphPad Software, La Jolla, CA, USA). *P*-values <0.05 between different experimental groups were considered significant.

## Results

### Expression of biomarkers of hypoxia in cultured osteoarthritis osteoblasts

**Figure 1:**
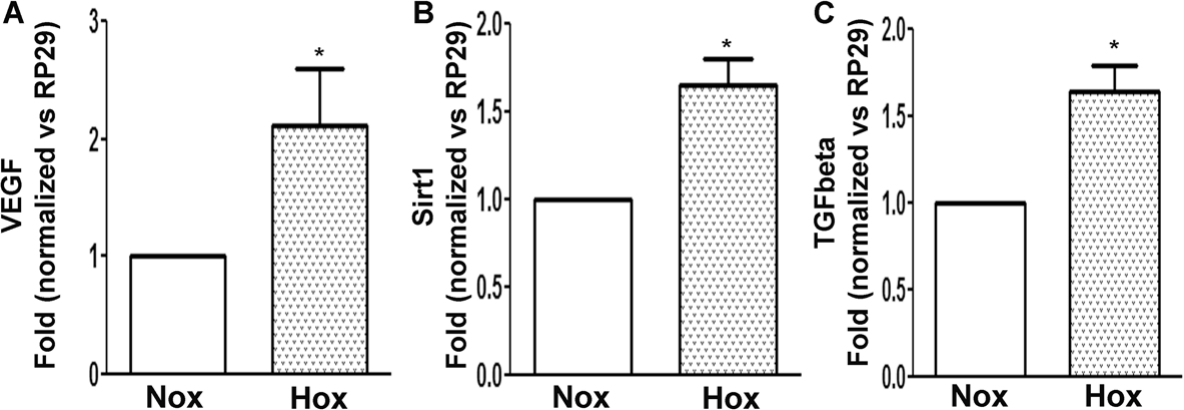
**Alteration of the expression of selective gene targets under hypoxia.** Human osteoarthritis (OA) osteoblasts were incubated for 24 hours either under normoxia (Nox) or hypoxia (Hox; 2% O_2_). Vascular endothelial growth factor (*VEGF*) **(A)**, *Sirt1* **(B)** and transforming growth factor β1 (*TGFbeta*) **(C)**. *P <0.05, statistically significant compared to Nox (*n* =4 to 6 experiments).

We first sought to determine whether hypoxia would affect OA Obs by evaluating the expression of selective gene targets. Figures 1A to 1C show that our experimental conditions stimulated expression of genes known to be induced by hypoxia (namely, *VEGF* (*P* <0.05) (Figure 1A), *Sirt1* (*P* <0.05) (Figure 1B) and *TGF-β1 P* <0.05) (Figure 1C)), thus validating their relevance to hypoxia.

**Figure 2:**
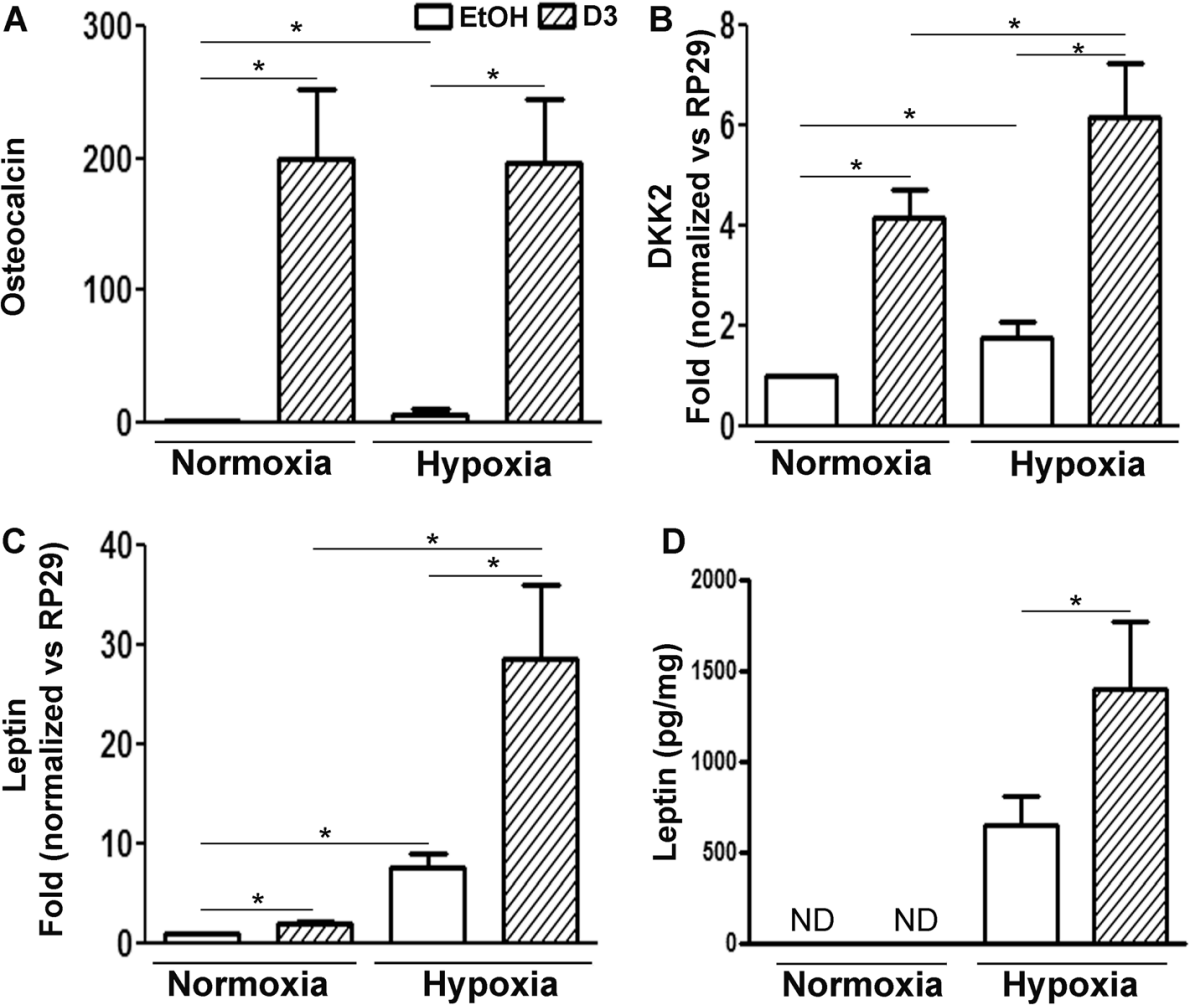
**Osteocalcin, dickkopf-related protein 2 and leptin expression under hypoxic conditions.** Human osteoarthritis osteoblasts were incubated for 24 hours (RT-PCR experiments) to determine the expression of osteocalcin **(A)**, dickkopf-related protein 2 (DKK2) **(B)** and leptin **(C)** (n =7 to 10 experiments), or for 48 hours (enzyme-linked immunosorbent assay experiments) to measure leptin production **(D)** (n =8 experiments) under either normoxia or hypoxia (2% O_2_) in the presence of 50 nM 1,25-dihydroxyvitamin D_3_ (D3) or vehicle (0.1% ethanol; EtOH). *P <0.05. ND, Not detectable.

### Expression of DKK2 and leptin in osteoarthritis osteoblasts under hypoxia

We next tested whether human OA Obs would still be responsive to VitD_3_ under hypoxia by analysing osteocalcin (OCN) expression, a late marker gene of Ob differentiation, by real-time RT-PCR (*n* =9). VitD_3_ increased OCN expression about 200-fold under both normoxia (*P* <0.05) and hypoxia (*P* =0.003) without any significant differences after 24 hours (Figure 2A). Of note, however, is that, in the absence of VitD_3_, hypoxia alone stimulated the expression of OCN about sixfold (*P* <0.05). We next evaluated whether hypoxia would lead OA Obs to produce variable levels of DKK2 (Figure 2B) and leptin (Figures 2C and 2D), which have been related to OA pathogenesis in bone. Hypoxia stimulated the expression of DKK2 only 1.8-fold (*n* =7, *P* <0.05), whereas VitD_3_ stimulated the expression of DKK2 about 4-fold under normoxia (*P* <0.05) and 3-fold under hypoxia (*P* <0.02). Hypoxia stimulated the expression of leptin sevenfold (*n* =10, *P* <0.05). Compared to control (hypoxia without VitD_3_), VitD_3_ stimulated leptin expression twofold under normoxic conditions (*P* <0.05) and fourfold under hypoxia (*P* <0.03) (Figure 2C). Hence, the combined effect of VitD_3_ and hypoxia stimulated leptin and DKK2 production 28- and 6.2-fold, respectively, compared to control under normoxia. As hypoxia was more powerful in enhancing leptin expression in OA Obs, we next evaluated the capacity of OA Obs to synthesize leptin. When we assessed leptin production in media conditioned for 48 hours, we found no detectable level under normoxia without concentrating the conditioned media, whereas hypoxia led to detectable leptin production (Figure 2D). This hypoxia-induced production was further stimulated twofold with VitD_3_ (*P* <0.02) (Figure 2D).

**Figure 3:**
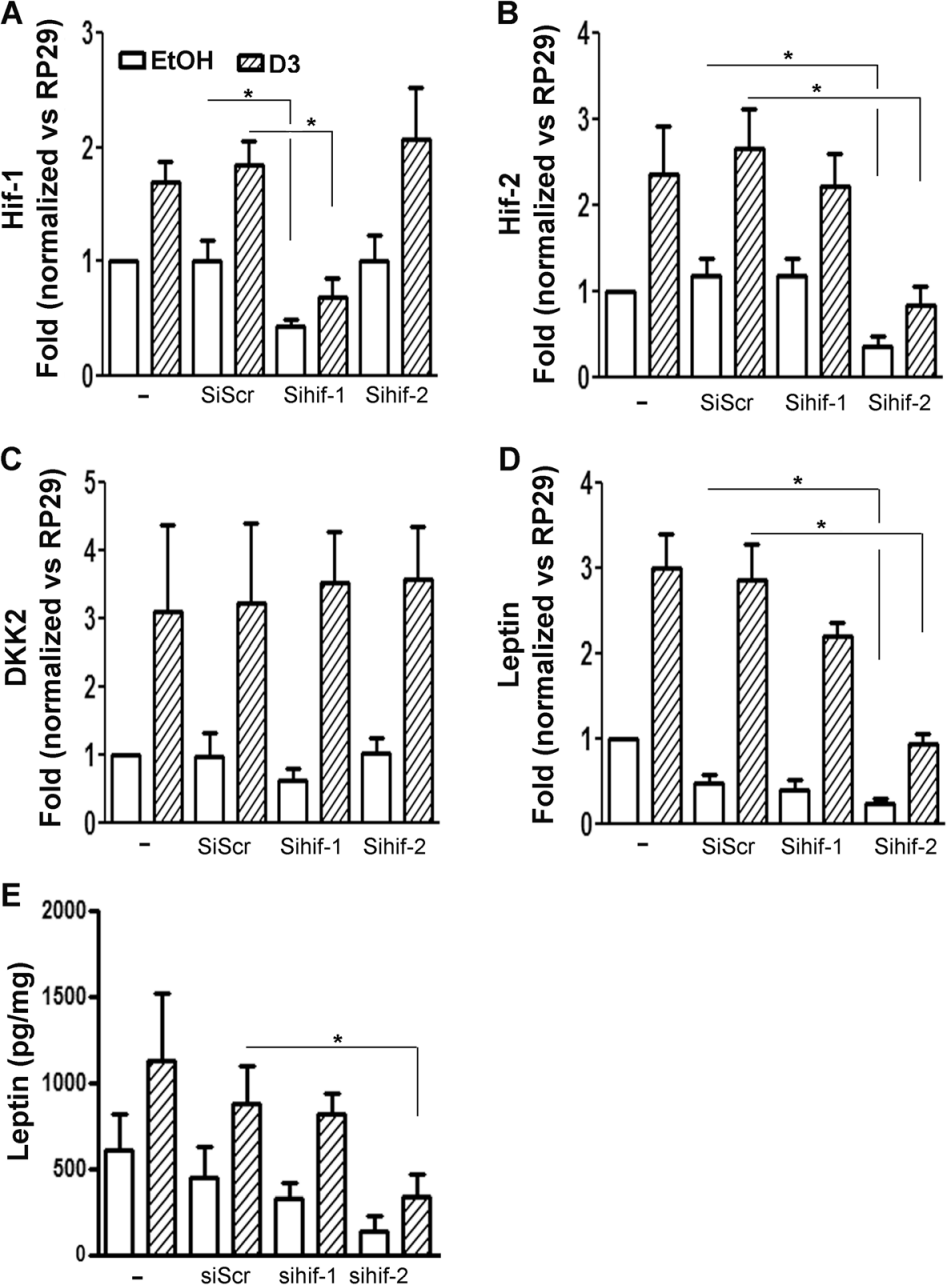
**Role of Hif-1 and Hif-2 in dickkopf-related protein 2 and leptin expression during hypoxia.** After following the RNA silencing protocol described in the Methods section, human osteoarthritis osteoblasts were incubated for 24 hours (RT-PCR experiments) to determine the expression of hypoxia-inducible factor 1 (Hif-1) **(A)**, Hif-2 **(B)**, dickkopf-related protein 2 (DKK2) **(C)** and leptin **(D)** (*n* =5 experiments), or for 48 hours to measure leptin production **(E)** (*n* =5 experiments), under hypoxia in the presence of 50 nM 1,25-dihydroxyvitamin D_3_ (D3) or 0.1% EtOH. SiSrc, Scrambled silencing RNA (control). *P <0.05.

### Regulation of the expression of DKK2 and leptin in osteoarthritis osteoblasts under hypoxia using siHif-1α and siHif-2α

We first verified that siHif-1α and siHif-2α decreased the expression of their respective target genes under hypoxia using RT-PCR (*n* =5). Compared with siScr conditions, siHif-1α decreased its own expression by almost 60% under basal conditions (*P* <0.03) and with VitD_3_ stimulation (*P* <0.04) (Figure 3A) without affecting Hif-2α mRNA levels. Under both conditions, siHif-2α decreased its own expression by 70% compared to the level of siScr (*P* <0.006 for basal conditions and *P* <0.008 with VitD_3_ stimulation) (Figure 3B) without affecting Hif-1α expression. We next evaluated whether DKK2 and leptin expression would be modified under hypoxia using siHif-1α and siHif-2α. Neither siScr, siHif-1α nor siHif-2α had any significant effect on the expression of DKK2 (Figure 3C). Leptin expression was slightly, but not significantly, downregulated in the presence of siScr under basal conditions, whereas it remained unchanged in the presence of VitD_3_ (Figure 3D). No significant inhibition of leptin expression was observed using siHif-1α alone, although a trend towards a decrease was noticed. Conversely, siHif-2α inhibited leptin expression by 50% compared to siScr (*P* <0.05) under basal conditions and by 67% with VitD_3_ incubation (*P* =0.004) (Figure 3D). We next verified the impact of Hif silencing on leptin protein levels. After 48 hours under hypoxia, no significant differences in leptin production were observed between siScr conditions or without any siRNA in the absence of VitD_3_, although a trend towards decreasing leptin levels was observed with siHif-2α (Figure 3E). Conversely, in the presence of VitD_3_, siHif-2α decreased leptin production significantly by 60% compared to siScr (*P* <0.02), whereas siHif-1α did not.

**Figure 4:**
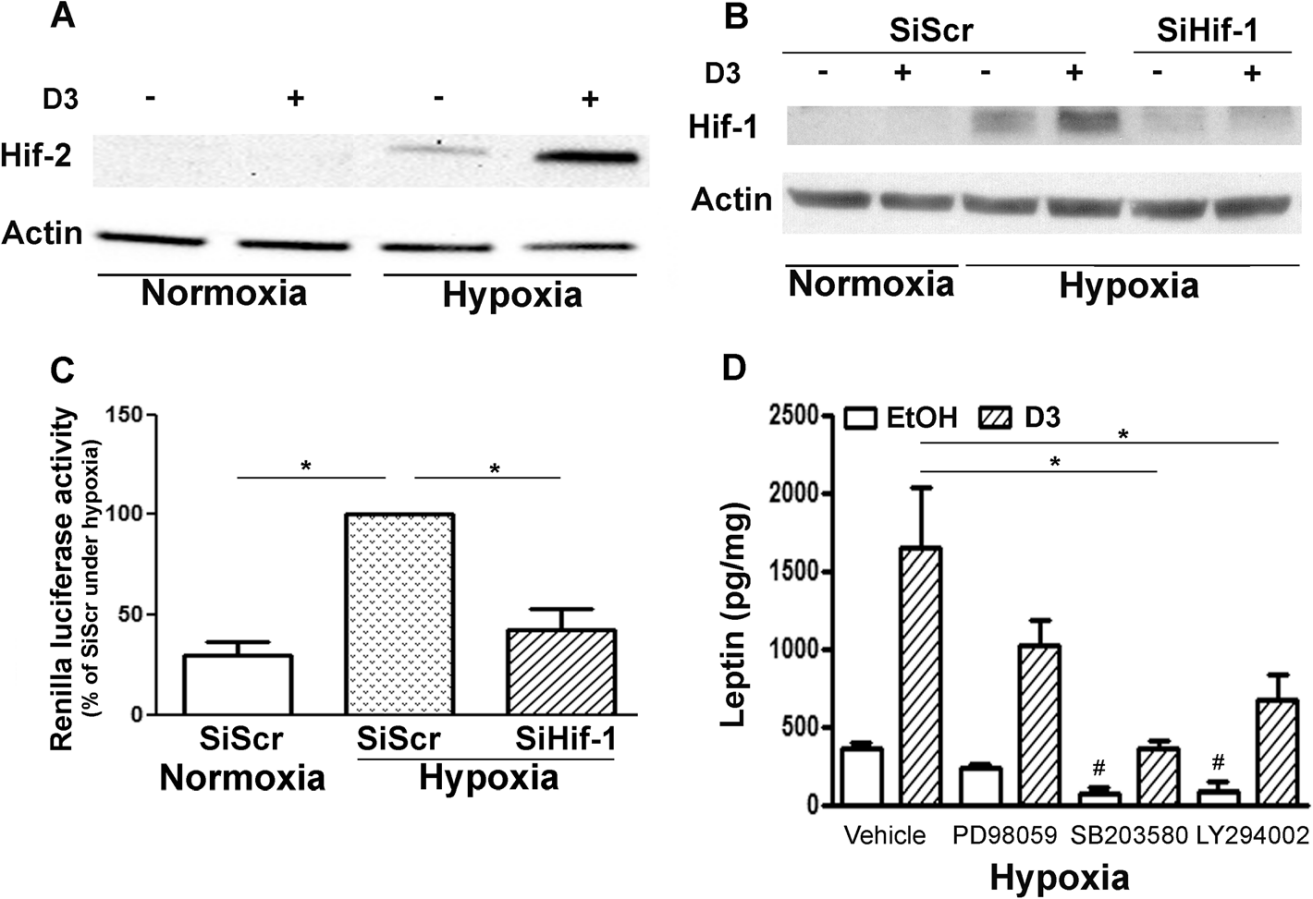
**Signalling pathways of hypoxia- and 1,25-dihydroxyvitamin D**_**3**_ **-stimulated leptin production. (A)** Cells were treated with EtOH (−) or 1,25-dihydroxyvitamin D_3_ (VitD_3_; +) and immediately incubated under normoxia or hypoxia. Immunoblot illustrating hypoxia-inducible factor 2 (Hif-2) stabilization representative of three independent experiments is shown. **(B)** Cells were treated with scrambled silencing RNA (siScr) or siHif-1 for 24 hours, then with EtOH (−) or VitD_3_ (+) and immediately incubated under normoxia or hypoxia for 6 hours. Immunoblot illustrating Hif-1 stabilization representative of four independent experiments is shown. **(C)** Cells were treated with siScr or siHif-1 for 24 hours, then transfected with a Hif-1 response element driving Renilla luciferase gene and incubated under hypoxia for 36 hours. *P <0.05. **(D)** Cells were pretreated with vehicle (0.1% dimethyl sulphoxide) or different inhibitors for 1 hour under normoxia, then EtOH or VitD_3_ was added and cells were immediately incubated under hypoxia for 48 hours (*n* =5 experiments). PD98059, inhibitor of mitogen-activated protein kinase kinase (MEK); SB203580, p38 mitogen-activated kinase inhibitor; LY294002, Phosphoinositide 3-kinase inhibitor. *P <0.05. ^#^Significant compared to vehicle (EtOH).

### Signalling pathways involved in leptin production

According to the above-described results, VitD_3_-induced leptin production was regulated mainly by Hif-2α. Therefore, we next investigated whether VitD_3_ could increase this protein in Obs. As expected, we found by immunoblotting that hypoxia increased Hif-2α stabilization. Moreover, this stabilization was greatly increased in the presence of VitD_3_ (Figure 4A) and peaked after 6 hours of hypoxia (kinetics not shown). Although Hif-1 does not seem to be involved in leptin production, we investigated whether this factor was also activated. Our results demonstrated that hypoxia also stabilized Hif-1 and that this stabilization was slightly enhanced by VitD_3_, but was prevented in the presence of siHif-1 (Figure 4B). In addition, by using a Hif-1α response element construction, we discovered that the stabilized Hif-1 protein was active under our experimental conditions of hypoxia, whereas treatments with siHif-1 decreased the hypoxia-induced Renilla luciferase activity (Figure 4C). Thus, these results reinforce those shown in Figure 3, indicating that, although Hif-1α was active, Hif-2 was responsible for the increase in leptin under hypoxia.

Next, using selective kinase inhibitors, we investigated whether pathways other than Hifs could be involved in leptin production. Inhibition of p38 MAPK and PI3K for 48 hours under hypoxia significantly decreased both basal (*P* <0.001 and *P* <0.006, respectively) and VitD_3_-dependent leptin production by OA Obs (*P* <0.03 and *P* <0.05, respectively). Inhibition of the p40/42 kinase pathway by PD98059 marginally decreased leptin production without VitD_3_ (*P* <0.02), but had no significant effect when VitD_3_ was present (Figure 4D).

### Search for crosstalk between leptin and DKK2

**Figure 5:**
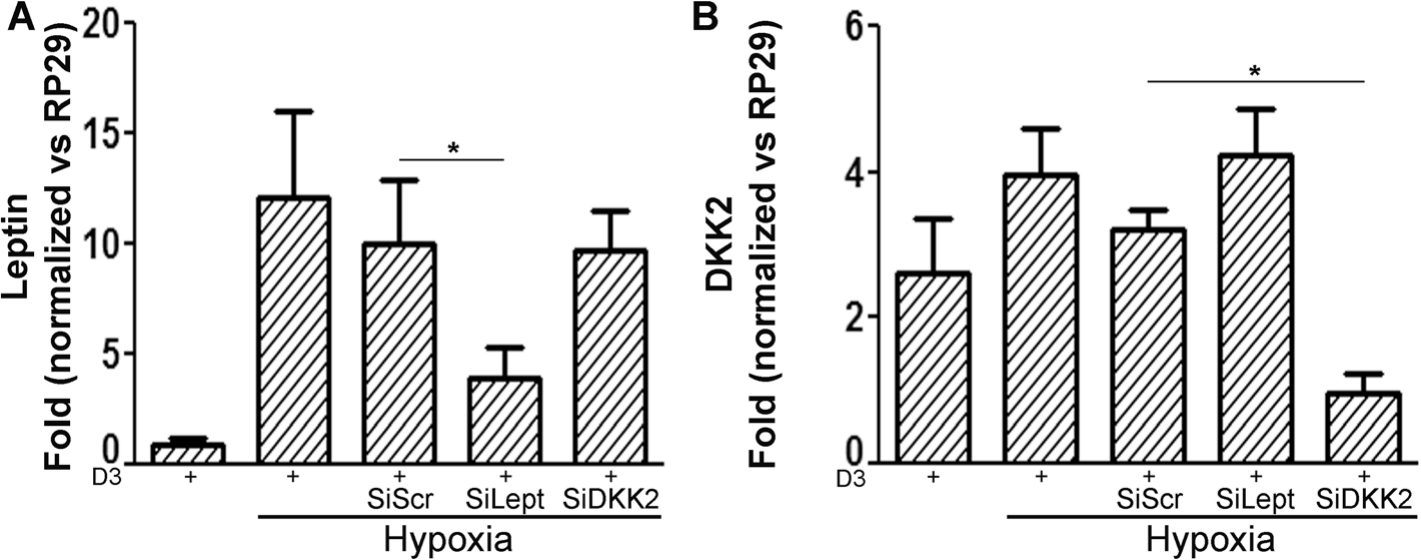
**Link between leptin and dickkopf-related protein 2 expression under hypoxia.** Human osteoarthritis osteoblasts were incubated for 24 hours under hypoxia (2% O_2_) in the presence of 50 nM 1,25-dihydroxyvitamin D_3_ (D_3_) to optimize leptin and dickkopf-related protein 2 (DKK2) production (*n* =5 or 6 experiments). The RNA silencing protocol described in the Methods section was followed for siDKK2 and siLept. *Statistically significant vs siScr D_3_. siScr, Scrambled silencing RNA.

We next evaluated if the expression of leptin and DKK2 could be linked under hypoxia. Inhibiting leptin expression with siLept (*P* <0.03) (Figure 5A) failed to reduce the expression of DKK2 (Figure 5B). Similarly, inhibiting DKK2 with siDKK2 reduced the expression of DKK2 (*P* <0.001) (Figure 5B), but had no effect on leptin expression (Figure 5A).

## Discussion

In the present study, we show that human OA Obs produced higher amounts of leptin under hypoxia than under normoxia. Production leptin was further increased in the presence of VitD_3_. In addition, *DKK2*, another gene with disturbed expression in OA, was upregulated under hypoxia. The regulation of leptin was mainly Hif-2α-dependent, because the knock-down of Hif-1α expression failed to affect leptin expression. Hypoxic regulation of leptin is also under the control of p38 MAPK and PI3K signalling pathways. Unlike leptin, the direct regulation of DKK2 seemed to be independent of either Hif-1α or Hif-2α. Also, under hypoxia, leptin did not alter the expression of DKK2, whereas, reciprocally, DKK2 did not alter the expression of leptin. This indicates that hypoxia *per se* was responsible for their respective increase and that neither leptin nor DKK2 can regulate each other.

Vascularization was reported to be increased in the subchondral bone plate during early to late stages of OA [7,8]. In other studies, researchers found that bone hypertension and vascular stasis generated hypoxia in OA [11,12,20]. Interestingly, in the spontaneous OA guinea pig model, changes in perfusion outflow of gadolinium with diethylenetriaminepentaacetic acid at 6 to 9 months temporally preceded and spatially colocalized with both cartilage degradation and bone remodelling in the medial tibial plateau [12]. Measuring real partial oxygen pressure in bone is difficult to assess, and concentrations of 3% to 7% have been described [45,46]. Meanwhile, Hif signalling has been demonstrated to intervene during both normal bone physiology and pathologies, indicating that values under 3% can be reached [23]. These hypoxic conditions could thus explain why OA Obs produce higher levels of VEGF [47] and hepatocyte growth factor [48], particularly in the sclerotic zones [49]. Consistent with these findings, our results show that hypoxia directly regulates VEGF expression by OA Obs (Figure 1A). Nevertheless, hypoxia could modify the phenotype of OA Obs [21], and, as described previously, Hif-1α and Hif-2α are the main effectors in the modulation of gene transcription under hypoxic conditions [23].

Regulation of the activity of these factors is complicated because their transcriptional regulation is not fully predictive of their protein level. For example, in the present study, 24-hour incubation with VitD_3_ induced Hif-2α expression by almost twofold under normoxia (data not shown); however, protein stabilization was almost undetectable in immunoblot analysis (Figure 4A). However, after 6 hours of hypoxia, Hif-2α protein was strongly increased in the presence of VitD_3_. Similarly, we observed that VitD_3_ increased Hif-1α expression. It was not involved in leptin regulation, however. VitD_3_ has been reported to be involved in the regulation of Hif-1α in breast epithelial cells [50], but, to our knowledge, the present study is the first one demonstrating the regulation of Hif-2α by VitD_3_. Among potential genes regulated by hypoxia, we paid attention to leptin, which plays a role in the pathogenesis of OA [29,33]. In preliminary experiments, we found that leptin expression was similar at 20% and 12% oxygen tension, followed by a noticeable increase at 7% and a huge stimulation at 3% and 2% (data not shown). Leptin production was also synergistically regulated by hypoxia and VitD_3_, and Hif-2α was the main regulator of leptin production under hypoxia. Conversely, Hif-1α played almost no role in leptin regulation in OA Obs. This situation has also been observed in fibroblast-like synoviocytes in rheumatoid arthritis [37]. However, the alternate regulation by either Hif-1α or Hif-2α factors might be species-dependent, because leptin is regulated mainly by Hif-1α under hypoxic conditions in zebrafish [51].

With regard to the leptin promoter, we found a hypoxia-responsive element (HRE) site juxtaposed to a potential vitamin D–responsive element (VDRE) site, which allows hypothesizing a theoretical cooperative interaction between VDRE and Hif-2α in a VDRE partnering model, as proposed by Carlberg and Campbell [52]. Therefore, VitD_3_ could be involved at two levels of leptin regulation, first by stabilizing Hif-2α protein levels and second by directly interacting with the VDRE present in the leptin promoter to synergistically stimulate leptin transcription. However, the presence of HRE and VDRE in the leptin promoter is probably necessary but not sufficient to presume a potential activation of transcription, because no synergy of transcriptional activity was noticed in other cell types [36].

We observed that leptin production is strongly regulated via p38 MAPK and PI3K, although we have not discriminated whether this regulation directly affected Hif-2α phosphorylation or targeted other factors. The role of leptin in bone metabolism is controversial [53,54]. However, in the context of hypoxia, which is known to induce osteocyte apoptosis within a few hours [55,56], the need for bone remodelling is crucial to maintaining bone health. Therefore, the coactivation of leptin expression by hypoxia and VitD_3_ should be considered a warning signal (that is, Obs sense oxygen tension), which, when oxygen tension decreases too drastically, alarms Obs to activate genes able to promote bone and vascular remodelling. In this context, leptin, which has been shown to increase Ob number and activity [29,57], as well as osteoclast activity [54,58], could play a role in remodelling. This type of scenario could happen, given the presence of defective capillaries within the subchondral bone plate, as reflected by the presence of stasis or microemboli, and could potentially lead to OA if the vasculature is not fully repaired. This would explain why Obs produce high levels of VEGF in response to hypoxia [47], as we show in the present study, leading to a compensatory hypervascularization of the subchondral bone that might not, however, result in fully mature functional vessels. If we assume that vasculature dysfunction plays a role in OA [20], this could explain the high prevalence of OA in patients who show signs of the metabolic syndrome, in whom the vascular system is often affected. If the vasculature fails to be well repaired, sustained amount of leptin could then be produced in the vicinity of the defect, which would lead over time to impairments in leptin signalling, as observed in obese patients [59]. Therefore, the effects of both the anabolic and catabolic properties of leptin on bone cannot be fully achieved, leading to (1) an uncoupling of bone remodelling and, possibly, to sclerosis of the subchondral bone plate and (2) an abnormal OA Ob phenotype, as previously described [29]. In addition to the overproduction of leptin, a combination of hypoxia and VitD_3_ increased DKK2 expression, which has also been related to the abnormal phenotype of OA Obs [31]. Unlike leptin, the expression of DKK2 was not significantly repressed by either Hif-1α or Hif-2α. Hence, as neither siHif-1α nor siHif-2α altered the expression of DKK2, which was stimulated under hypoxia, yet another mechanism must be set in place to regulate the expression of DKK2 under hypoxia. However, we and others previously demonstrated that transforming growth factor β1 (TGF-β1) can promote the expression of leptin in human Obs and rat bone tissue [29,30], as well as the expression of DKK2 in human primary Obs [31]. We also observed that hypoxia was able to induce TGF-β1 expression in OA Obs, which could explain, in part, the links between hypoxia, TGF-β1, leptin and DKK2 and why the expression of DKK2 could not be directly modulated by siHifs. However, the intricate relationship between these factors remains to be fully investigated.

## Conclusion

We demonstrate a synergism between hypoxia and VitD_3_ in leptin production by human OA Obs, suggesting that problems in the vasculature of the subchondral bone could play a role in the development of OA. In this study, we produced new data confirming the hypothesis of a recently published study [21] in which the researchers found that hypoxia modulates the Ob phenotype. Vascularization of the subchondral bone plate during OA needs to be explored more in depth to gain understanding of the role of oxygenation in the physiopathology of bone remodelling in OA.

## Abbreviations

DKK2: Dickkopf-related protein 2
ELISA: Enzyme-linked immunosorbent assay
FBS: Foetal bovine serum
Hif: Hypoxia-inducible transcription factor
HRE: Hypoxia-responsive element
MAPK: Mitogen-activated protein kinase
MRI: Magnetic resonance imaging
OA: Osteoarthritis
Ob: Osteoblast
OCN: Osteocalcin
PI3K: Phosphoinositide 3-kinase
siRNA: Silencing RNA
siScr: Scramble silencing RNA
TGF-β1: transforming growth factor β1
VDRE: Vitamin D–responsive element
VEGF: Vascular endothelial growth factor
VitD_3_: 1,25[OH]_2_ vitamin D_3_
